# Niche divergence builds the case for ecological speciation in skinks of the *Plestiodon skiltonianus* species complex

**DOI:** 10.1002/ece3.1610

**Published:** 2015-10-05

**Authors:** Guinevere O. U. Wogan, Jonathan Q. Richmond

**Affiliations:** ^1^ Museum of Vertebrate ZoologyIntegrative BiologyUniversity of California, Berkeley3101 Valley Life Sciences BuildingBerkeleyCA94720; ^2^ U.S. Geological SurveyWestern Ecological Research Center4165 Spruance Rd. Suite 200San DiegoCA92101‐0812

**Keywords:** Adaptation, environmental niche models, niche overlap, phenotype–environment association, thermal adaptation

## Abstract

Adaptation to different thermal environments has the potential to cause evolutionary changes that are sufficient to drive ecological speciation. Here, we examine whether climate‐based niche divergence in lizards of the *Plestiodon skiltonianus* species complex is consistent with the outcomes of such a process. Previous work on this group shows that a mechanical sexual barrier has evolved between species that differ mainly in body size and that the barrier may be a by‐product of selection for increased body size in lineages that have invaded xeric environments; however, baseline information on niche divergence among members of the group is lacking. We quantified the climatic niche using mechanistic physiological and correlative niche models and then estimated niche differences among species using ordination techniques and tests of niche overlap and equivalency. Our results show that the thermal niches of size‐divergent, reproductively isolated morphospecies are significantly differentiated and that precipitation may have been as important as temperature in causing increased shifts in body size in xeric habitats. While these findings alone do not demonstrate thermal adaptation or identify the cause of speciation, their integration with earlier genetic and behavioral studies provides a useful test of phenotype–environment associations that further support the case for ecological speciation in these lizards.

## Introduction

Ecological speciation occurs when natural selection causes divergence in traits that influence reproductive compatibility, eventually leading to a cessation of gene flow between lineages that have adapted to different environments (Dobzhansky 1937; Mayr 1942; Schluter 2000). Demonstrating the process requires the identification of traits under divergent selection, and that those same traits are responsible for reproductive isolation (Rundle and Nosil 2005; Funk 2009; Nosil 2012). Of the relatively few well‐defined examples of ecological speciation that exist, trait evolution in response to host, pollinator, and diet shifts has been causally linked to reproductive isolation (reviewed in Funk 2009; Nosil 2012). Thermal adaptation to different habitats has also been implicated as mechanism by which traits associated with physiological ecology may be linked to reproductive compatibility, but few studies have thoroughly explored this possibility (Keller and Seehausen 2012).

Worldwide, spatially explicit environmental data have been increasingly used to test for ecology's role in speciation, with comparisons of climate‐based environmental niche models between sibling species representing the key analytical framework (Graham et al. 2004; Kozak et al. 2008; Warren et al. 2008; McCormack et al. 2010). This approach represents one of several potential steps in demonstrating a role for thermal adaptation in the speciation process, given that an organism's optimal performance is expected to match the climate regime it experiences most often (Angilletta 2009; Keller and Seehausen 2012). Although tests of niche divergence and equivalency do not provide definitive measures of thermal adaptation or direct evidence on whether adaptive differences evolved before or after the establishment of reproductive isolation, they can be used to provide evidence for the outcomes of such a process. If a premating barrier is tied to divergence in traits with clear physiological implications, while at the same time niche divergence is demonstrated between sibling species with different trait values, then evidence is gained for ecological speciation.

**Figure 1:**
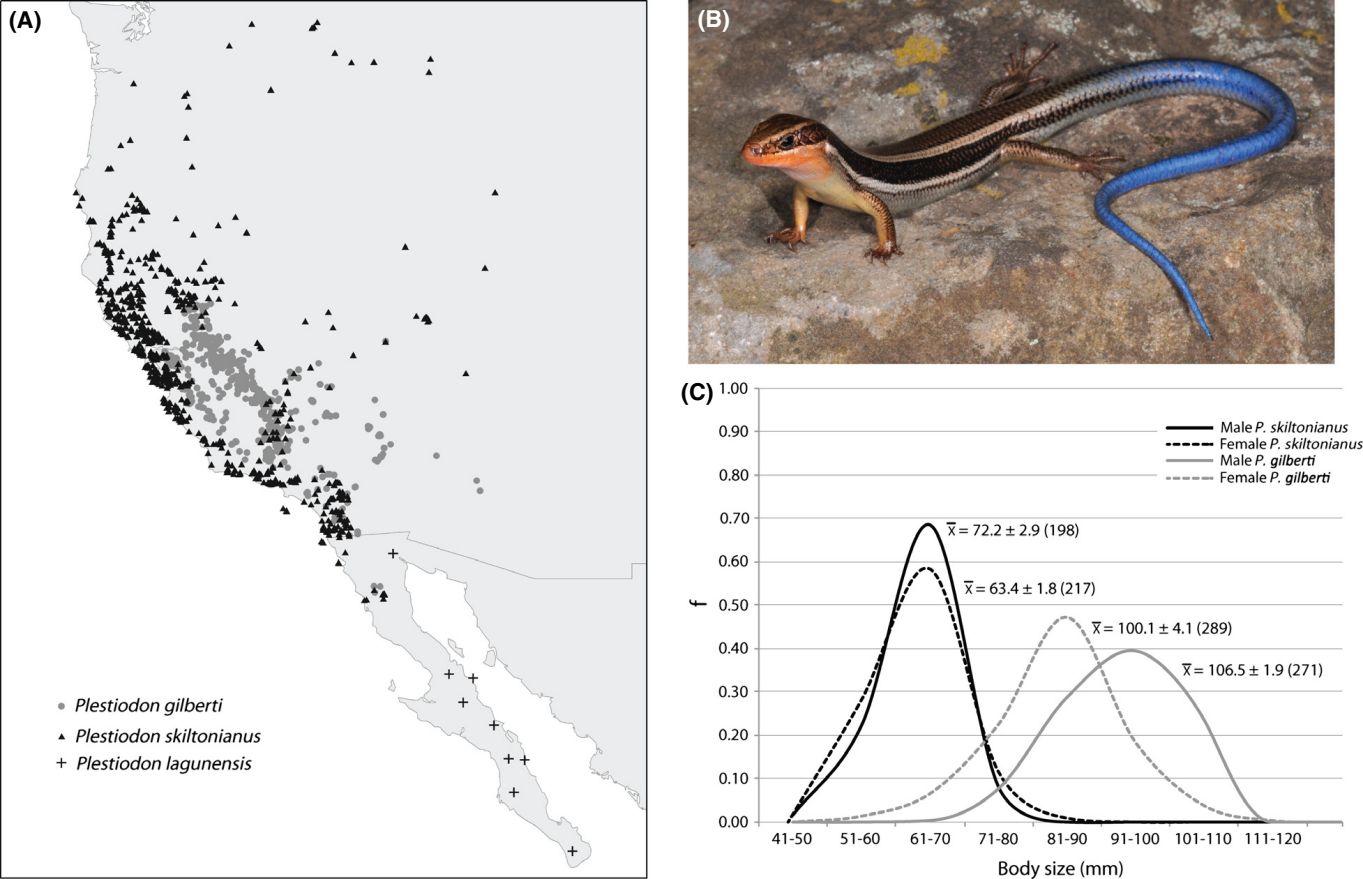
The three lizard species forming the *P. skiltonianus* complex fall into two distinctive morphospecies a large‐bodied, unicolored form and a geographically widespread, small‐bodied form. (A) Occurrence points for members of the *P*. *skiltonianus* complex. (B) Young individual of the small‐bodied form, *P. skiltonianus* (photograph by Jackson Shedd). (C). Body size distributions for species of the *P*. *skiltonianus* complex. Size bins on the *x*‐axis reflect snout‐vent lengths (SVL) for 415 specimens of *P*. *skiltonianus* and 560 *P*. *gilberti*. Mean SVL ± SD (no. of specimens) is provided for males and females of both species.

One candidate example of ecological speciation is the scincid lizards of the *Plestiodon skiltonianus* species group (Richmond and Reeder 2002). The group is widely distributed in western North America and is comprised of three nominal species: *P. skiltonianus*,* P. gilberti*, and *P. lagunensis* (Fig. 1). The three species fall into two general morphotypes – a large‐bodied (>85.0 mm snout‐to‐vent [SVL]) morph characterizes typical adult *P. gilberti*, whereas a small‐bodied form characterizes *P. skiltonianus* and *P. lagunensis* (almost always <80.00 mm SVL: Fig. 1). Most work on this group to date has focused on understanding the role of body size divergence in reproductive isolation, rather than the causes of the size divergence itself. No‐choice mating experiments have shown that copulation success is dependent on the size difference between paired individuals and that a mechanical incapability arising from the geometry of the mating posture is the main reproductive barrier causing nonrandom mating between morphospecies (Richmond and Jockusch 2007; Richmond et al. 2011). At least some molecular phylogenetic evidence also indicates that the mechanical barrier has evolved in parallel between multiple sister species pairs (Richmond and Jockusch 2007), a common signature of divergent natural selection (Schluter 2000); however, the evidence for parallelism within the *P*. *skiltonianus* group is based largely on mtDNA data, and preliminary analysis of whole genome, restriction‐associated digest sequences shows conflicting signals with respect to the number of times that the different morphotype pairs have evolved. Nevertheless, the fact remains that body size divergence is the cause of reproductive isolation, but no studies have addressed whether ecological differences might be responsible for the changes in size.

Anecdotal observations indicate that the larger‐bodied *P. gilberti* occurs in warmer and more arid environments relative to the small‐bodied species, which tends toward more mesic conditions and occurs over a much broader range of elevation and latitude. These associations are consistent with predictions of the “surface law” (Schmidt‐Nielsen 1984), where *P. skiltonianus* and *P. lagunensis* likely experience more rapid heat exchange and evaporative water loss across the skin due to an increased body surface area‐to‐volume ratio compared to the larger and stockier *P. gilberti*. Coupled with the fact that these skinks are sensitive to desiccation and tend to be active earlier in the year than most lizards (Richmond 2005), *P. gilberti* may have a higher tolerance for warmer and drier environments than either *P. skiltonianus* or *P. lagunensis* as a consequence of larger body size.

For ectotherms such as lizards, ambient climatic and thermal conditions influence a variety of physiological life history traits, including locomotion and sprint performance, reproductive strategy, and growth (Huey and Stevenson 1979; Angilletta et al. 2006; Deutsch et al. 2008; Angilletta 2009). Traits that relate to thermal or climatic tolerances are expected to be relatively conserved (Sunday et al. 2011; Keller and Seehausen 2012; Svensson 2012; Grigg and Buckley 2013), a prediction that is largely supported for lizards (Youssef et al. 2008; Araújo et al. 2013; Moreno Azócar et al. 2013; Muñoz et al. 2013). When thermal or climatic niches are highly conserved among taxa, speciation tends to be associated with allopatry (Wiens 2004; Kozak and Wiens 2006). However, when niche conservatism erodes, ecological divergence tends to be the culprit and speciation does not necessarily depend on allopatry (Svensson 2012). Svensson (2012) suggests that if thermal or climatic conditions do play a major role in ecological speciation, a logical outcome is that closely related species will have differentiated thermal or climatic niches.

In this study, we examine whether current distributions and habitat associations of members of the *P. skiltonianus* complex are consistent with reproductively isolated species that have diverged in different environments. Specifically, we use large‐scale climate data and null models to test whether the two morphospecies occupy equivalent niches, whether their respective niches differ significantly from one another after removing the effects of the background environment (i.e., location), and the role of the niche in determining species distributions. Using the two morphospecies as our basic unit of comparison, we explicitly test the association between body size and the environment. To do this, we develop a mechanistic niche model based on lethal physiological temperature (Monahan 2009) and a correlative model based on the maximum entropy approach (Phillips et al. 2006). We focused on comparisons between the two morphospecies rather than among lineages within the species complex because (1) size is the fundamental trait responsible for the mechanical reproductive barrier and (2) it is necessary to rule out niche conservatism between the two main morphs in order to set precedence for thermal adaptation as a promoter of ecological speciation. If ecological speciation has occurred within the *P. skiltonianus* complex, we predict that the two main morphospecies will have significantly divergent thermal and/or climatic niches and that niche divergence between morphs will be greater than expected given differences in the background environment.

## Methods

### Niches and niche models

Hutchinson (1957) defined the niche space as a p‐dimensional set of interacting variables, where different combinations of variables describe the conditions under which individuals are able to survive and reproduce. These combinations are referred to as the fundamental, potential, and realized niches. The fundamental niche represents the set of all possible environmental conditions and resources that a species can use in the absence of other organisms, given intrinsic limits to growth, survival, and reproduction. The potential niche corresponds to a subset of the fundamental niche and represents the portion of environmental space in which the species can survive and reproduce at a particular point in time (Jackson and Overpeck 2000). The realized niche in turn is a subset of the potential niche and represents the portion of environmental space actually occupied by the organism in nature, constrained by resources and other biotic factors (Figure S1). Distinguishing among the fundamental, potential, and realized niches is difficult due to the interrelationship among them, yet doing so is key to understanding the factors that shape species' distributions. For this study, we explore two types of niche models (mechanistic and correlative), each using different types of data that represent different aspects of the species niche.

Mechanistic niche models incorporate species‐specific physiological and life history data into distribution modeling and are presumed to be strong representatives of fundamental niche space (Kearney and Porter 2009). Thermal performance curves capture the direct effect of temperature on fitness by measuring the ability of an organism to perform physiological functions at different temperatures and are circumscribed by critical thermal limits (Deutsch et al. 2008; Angilletta 2009). Critical thermal physiological data were available for *P. skiltonianus* and *P. gilberti* (Brattstrom 1965; Cunningham 1966; Youssef et al. 2008), which covers the bulk of the morphological diversity in the *P. skiltonianus* complex. These measures mark the critical thermal limits of the fundamental niche. We used paired values of *T*_MEAN_ and *T*_MAX_ to calculate the hottest and coldest climatic extremes during months when the lizards are most active (roughly March–June) and then calculated the areas of the fundamental, potential, and realized niche using °C^2^ with precision of 0.1°C following the procedure of Monahan (2009) (additional details are provided in Supplemental Materials S1). From here forward, we refer to the niche estimated from these data as the thermal niche.

We generated correlative niche models for each species using the maximum entropy approach implemented in MaxEnt v. 3.3.2 (Phillips et al. 2006). MaxEnt requires only locality records and climate data to generate a species distribution and has been shown to be good approximation of mechanistic models and a reliable predictor of species distributions (Elith et al. 2006; Hijmans and Graham 2006). These models represent an approximation of the realized niche and assume that the realized and fundamental niches of a species coincide. We tested model accuracy using 25% of the occurrence locals reserved from model training, as well as the area under the receiver operating characteristic curve (ROC). We used jackknife statistics to identify environmental variables that were the best predictors of species' distributions. Contemporary models were generated using nineteen WorldClim bioclimatic layers at 30‐arc second resolution (Table S1) (Hijmans et al. 2005), and paleodistribution models were modeled for three time periods: the Holocene Hypsithermal (6 ky), the Last Glacial Maximum (21 ky); and the Last Interglacial Period (~120 ky) using the same 19 variables at the same resolution but derived from paleo‐GCMs (DKRZ, 1992; Otto‐Bliesner et al. 2006). Paleomodels were used only to estimate the stable range of each species, which was then used in calculating overlap ratios. We do not present these models in the text, but have provided them in S2 and Figure S2. From here forward, we refer to the niche estimated from these data as the climatic niche.

### Niche differentiation

If reproductive isolation evolved as a by‐product of thermal adaptation in the *P. skiltonianus* group, one outcome of the process is that the climate niches of the different morphospecies should be differentiated. We used Warren's identity test (Warren et al. 2008) to test whether the climatic niches of the *P. skiltonianus* group members are significantly divergent and whether they are more or less similar than expected by chance. This test uses the probability of occurrence from correlative models in combination with species' occurrence and climate data to calculate the similarity metrics *I* (Warren et al. 2008) and *D* (Schoener 1968). Values for both metrics range from 0 (no niche overlap) to 1 (identical niche) and are used to determine whether the niches of different species in different geographic areas are equivalent, and to measure how well the niche of one species' range predicts the niche of another compared to a randomly generated species niche from the same environment (Peterson et al. 1999; Warren et al. 2008).

A confounding factor in testing for differences in species' niche occupancy is spatial autocorrelation, whereby changes in environmental variables may simply reflect spatial gradients (i.e., the background environment; Legendre 1993; Dormann et al. 2007). We implemented analyses that correct for this problem by testing a null model in which species' niches differ in a manner that is consistent with changes in the background environment (McCormack et al. 2010). Deviations from the null model (i.e., *D*_back_ = *D*_obs_) can provide evidence of niche divergence (i.e., *D*_back_ < *D*_obs_) or niche conservatism (i.e., *D*_back_ > *D*_obs_; (McCormack et al. 2010)). We then performed the same series of niche equivalency and niche similarity tests using the method of Broennimann et al. (2011), which differs from the Warren and Schoener methods in that it relies directly on sampling points rather than correlative niche models to quantify niche overlap. The benefit of this method is that it accounts for spatial autocorrelation while correcting for issues related to the resolution of the environmental data and sampling variation across the species' distribution. We further quantified niche overlap among species using PCA and climate parameters obtained from smoothed occurrence data (PCA‐occ) and from the entire environmental space covered by the species' ranges (PCA‐env; Broennimann et al. 2011).

Last, we used classic multivariate statistical methods (MANOVA, DFA) to quantify differences in the mean values of environmental variables among species (additional details are provided in the supplemental methods S1).

### Niche dynamics and distributions

A prediction of ecological speciation by adaptation to different thermal environments is that species' distributions should reflect differences in their critical thermal limits. To test the extent to which critical thermal limits are governing the distributions of the different *P. skiltonianus* group members (vs. biotic interactions or historical contingencies), we used a method that compares different ratios for the areas encompassed by the realized (*R*), potential (*P*), and fundamental (*F*) thermal niches (Monahan 2009). These areas are expected to be equal when the species' distributions are shaped solely by critical temperatures and unequal when other factors such as competition with other species, physical dispersal barriers, or other physiological constraints either interact with or override temperature. Using the areas of *R*,* P*, and *F* as measured in °C^2^, we calculated *P*/*F* and *R*/*P* on a scale from zero (maximum discord) to one (maximum congruence). If *P*/*F* is less than one, the species' critical temperatures cover a wider area of thermal niche space than is available given the current climate. *R*/*P*‐values less than one indicate that factors other than critical temperature are preventing the species from occurring in thermally suitable areas.

To quantify overlap between the potential climatic niche and the realized distribution for each species pair, we calculated the realized/potential range size ratio (or range‐filling ratio, *R*_rs_/*P*_rs_; Svenning and Skov 2004). This ratio uses the correlative models to estimate the realized and potential niches. It provides information on the extent to which a species fills its potential range and can assist in determining whether niche conservatism, niche divergence, competitive interactions, dispersal limitations, or other factors are influencing distribution limits (Costa et al. 2008; Figure S1).

## Results

### Niche models

**Figure 2:**
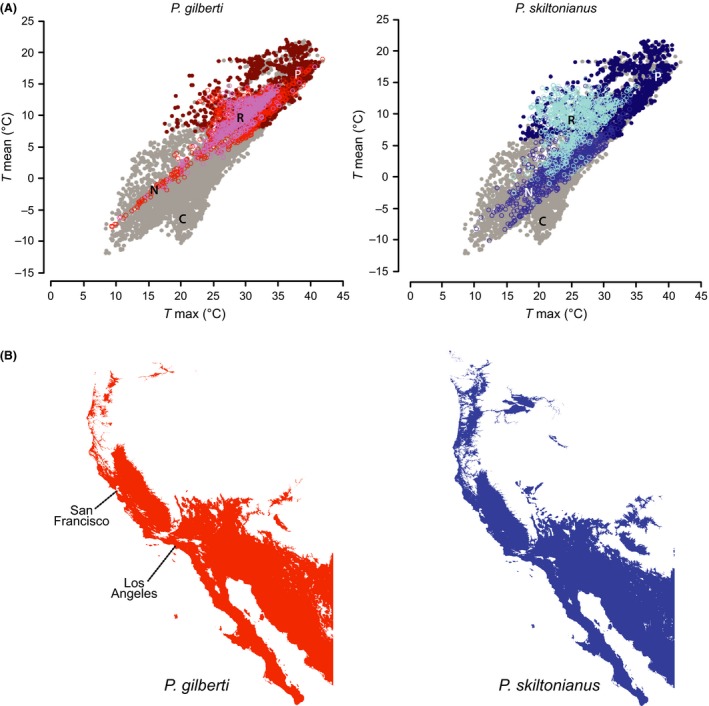
(A) Thermal niche space of *P*. *gilberti* and *P*. *skiltonianus* based on mechanistic models that incorporate thermal constraints during the adult active period (March to June). These panels depict the intersection of the realized climate (C, solid dark gray dots), the potential niche (P, solid dark red or solid dark blue), the IUCN delimited realized niche (N, open red or open medium blue), and the specimen delimited realized niche (R, open circles pink or pale blue). (B) The spatial distribution of the potential niche for each species.

The mechanistic niche models based on lethal physiological temperatures for *P. gilberti* and *P. skiltonianus* identified largely congruent fundamental and potential thermal niches. The primary differences in the potential thermal niche are in the deserts of southwestern North America and the mesic regions of the Pacific Northwest (Fig. 2). The realized thermal niche of the two species differs, with *P. gilberti* and *P. skiltonianus* occupying different portions of their congruent fundamental and potential thermal niche spaces (Fig. 2).

**Figure 3:**
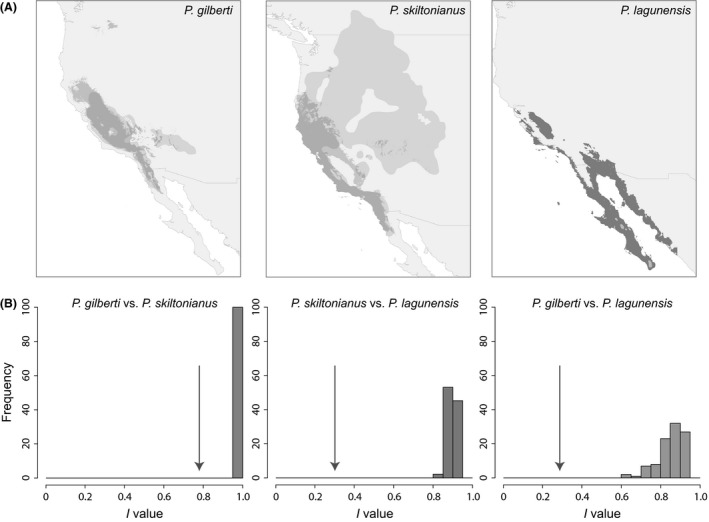
(A). Predicted species' distributions based on correlative ecological niche models (dark gray); the ranges have been bounded using equal sensitivity and specificity. Each panel depicts the predicted distribution under the present climate. The IUCN range for each species is also provided (light gray). (B) Species identity tests. Each graph depicts the distribution generated from 100 pseudoreplicated MaxEnt runs, which pool and then parse the presence points randomly. The arrow indicates the actual niche overlap score between pairwise niche comparisons among species. The niche overlap value is smaller than the null distribution in all pairwise comparisons, indicating that the niches have diverged.

The correlative models under current climatic conditions reveal distributions that generally match the realized distributions, with a few exceptions (Fig. 3). First, models predict that *P. gilberti* should be more widespread on California's Great Central Valley floor (hereafter Central Valley), including areas well north of the present‐day distribution. For *P. skiltonianus,* the predicted distribution is substantially more limited than the realized space, especially in the Sierra Nevada and the eastern part of the range outside of California. Like *P. gilberti,* the predicted distribution for *P. lagunensis* extends further north than the observed distribution, with suitable climatic space identified in the Central Valley and along much of coastal California. It is also predicted to have a more extensive range within Baja and mainland Mexico.

Maxent models for contemporary climate had AUC scores above 0.95 (Table 1) and therefore had high predictive accuracy. Precipitation variables primarily delimit the current distributions (Table 1). When compared to the IUCN distributions, the correlative niche models predict larger ranges for *P. gilberti* and *P. lagunensis*, and a smaller range for *P. skiltonianus* (Fig. 3, Table 2).

**Table 1:** AUC scores for contemporary correlative niche models. Results from jackknife analyses are given: The three bioclimatic variables that had the highest contribution and the percentage of the contribution are provided

Taxon	AUC	Variable 1	Variable 2	Variable 3
*P*. *gilberti*	0.966	18 (43)	15 (19.3)	14 (10.4)
*P*. *skiltonianus*	0.951	15 (33.3)	18 (30.2)	19 (15.9)
*P*. *lagunensis*	0.958	11 (57.4)	14 (18.8)	17 (9.3)

**Table 2:** Overlap of predicted species' ranges. The diagonal represents the current predicted distribution for each species (km^2^, the current distribution estimated from IUCN range polygons is given in parentheses). Above the diagonal – the percent of the combined predicted ranges where overlap is predicted between species; area of the predicted overlap is given in km^2^ in parentheses. Below the diagonal – percent overlap between species calculated from current distribution ranges; area of overlap predicted from the stable “core” range is given in km^2^ in parentheses

	*P. gilberti*	*P. skiltonianus*	*P. lagunensis*
*P. gilberti*	274,628 (216,398)	4.45% (174236)	5.49% (35547)
*P. skiltonianus*	6.59% (90395)	362,724 (115,5742)	0.04% (35926)
*P. lagunensis*	(0%)	(0%)	376,586 (3920)

### Niche differentiation

The identity test statistic *I* indicates that species climatic niches have diverged to a greater extent than predicted by null models of background environmental divergence. In all pairwise comparisons, climatic niche overlap values were significantly smaller than the null distribution, supporting a pattern of niche divergence rather than niche conservatism (Fig. 3, Table 3). Schoener's *D* statistic (based on correlative niche models) reveals that the climatic niche of *P. lagunensis* is highly divergent from both *P. gilberti* (0.126) and *P. skiltonianus* (0.127), whereas the climatic niches of the latter two species show a greater degree of overlap (0.5230; Table 3).

**Table 3:** ENM species overlap scores. The identity scores *I* are above the diagonal; Schoener's *D* score are below the diagonal

	*P. gilberti*	*P. skiltonianus*	*P. lagunensis*
*P. gilberti*	−	0.7965	0.3062
*P. skiltonianus*	0.5296	−	0.2723
*P. lagunensis*	0.126	0.1273	−

In nine of 11 instances, McCormack's test showed that the degree of climatic niche divergence among species was substantially greater than expected given the differences in the background environment, supporting niche divergence (Table 4). PC1 is related to temperature variables and PC2 is primarily related to precipitation variables (Table 5). The results from Broennimann's test revealed Schoener's *D* values ranging from 0.25 to 0.89 (Table 6). Niche equivalency tests found that seven of the 19 bioclim variables were equivalent among species, while the remaining 12 were significantly different (Table 6). Niche similarity tests failed to reject the null hypothesis of similarity in all but three climate measures: annual precipitation (bio12), precipitation of the wettest month (bio16) and precipitation of the coldest quarter (bio19) when comparing *P. gilberti* to *P. skiltonianus* (Table 6). Schoener's *D* values based on all 19 bioclim variables in the PCA‐env and PCA‐occ analyses were 0.29 and 0.30, respectively (Tables 6 and 7), indicating very little overlap among the first two PCs. PC1 and PC2 explained 77.71% of the observed variation in PCA‐env, and 73.42% in PCA‐occ (Table 6). Analyses of thermal maxima and precipitation reveal that the niches of *P. gilberti* and *P. skiltonianus* are largely differentiated along these two climate parameters during the active months, while the niches are equivalent with respect to thermal minima (Table S2).

**Table 4:** Ecological divergence of the realized niche along among species. PCs greater than 1 were retained for analyses. Statistically significant differences in principal components scores as assessed by *t*‐tests are indicated with *. The 95% CI of the background divergence is given in parentheses. Niche divergence (D) is inferred if the realized niche divergence is greater than the background divergence. Niche conservatism (C) is indicated if the realized niche divergence is less than the background divergence. *PLGI*,* P. gilberti*;* PLSK*,* P. skiltonianus*;* PLLA*,* P. lagunensis*

	PC1	PC2	PC3	PC4
*PLGI vs. PLSK*	0.479* **C** [2.448 − 2.680]	0.517* **D** [0.282 − 0.462]	1.124* **D** [0.045 − 0.147]	0.114* **D** [0.002 − 0.063]
*PLGI vs PLLA*	4.111* **D** [3.934 − 4.092]	2.260 [1.728 − 1.845]	3.059* **D** [1.821 − 1.893]	0.808* **D** [0.630 − 0.671]
*PLSK vs. PLLA*	4.589* **D** [1.373 − 1.539]	0.482* **C** [1.340 − 1.486]	1.934* **D** [1.724 − 1.801]	0.921* **D** [0.595 − 0.665]

**Table 5:** The bioclim variables delineated as important from all the niche differentiation analyses. For each PC (or DF), the variables are arranged from high to low based on loadings. Bioclim variables 1–11 concern temperature, while 12–19 concern precipitation. Refer to the Supplemental Results (Table S1) for details regarding each of the variables

	McCormack's test	Broennimann's test PCA‐env	Broennimann's test PCA‐occ	Multivariate (DFA)
PC1 (or DF1)	1, 11, 6	1, 10	1, 6	1, 12
PC2 (or DF2)	13, 16, 7	7, 4	4, 7	19, 13
PC3	18, 9, 19	Na	Na	Na
PC4	18, 17, 14	Na	Na	Na

**Table 6:** Model‐free assessment of niches using 19 bioclim variables. Schoener's *D* is a measure of niche overlap, and it scales from 0 (no overlap) to 1 (complete overlap). *P*‐values are given for both the niche equivalency and niche similarity tests. *P*‐values significant at the 0.05 level are in bold

Bioclim variables	Schoener's *D*	Niche equivalency	Niche similarity (2–1)	Niche similarity (1–2)
Bio1	0.782	0.2	0.1	0.38
Bio2	0.739	0.06	0.28	0.06
Bio3	0.489	**0.02**	0.53	0.08
Bio4	0.486	**0.02**	0.42	0.63
Bio5	0.746	**0.04**	0.34	0.91
Bio6	0.35	**0.02**	0.5	0.89
Bio7	0.492	**0.02**	0.67	0.63
Bio8	0.888	0.4	0.24	0.24
Bio9	0.385	**0.02**	0.32	0.85
Bio10	0.646	**0.02**	0.3	0.93
Bio11	0.784	0.22	0.14	0.32
Bio12	0.478	**0.02**	0.44	**0.04**
**Bio13**	0.537	**0.02**	0.3	0.06
Bio14	0.768	0.3	0.18	0.14
Bio15	0.25	**0.02**	0.4	0.42
Bio16	0.55	**0.02**	0.24	**0.04**
Bio17	0.796	0.53	0.14	0.2
Bio18	0.85	0.89	0.16	0.14
Bio19	0.563	**0.02**	0.42	**0.04**
PCA‐env	0.299	**0.02**	0.24	**0.02**
PCA‐occ	0.29	**0.02**	0.24	0.1

**Table 7:** The relationship between fundamental, potential, and realized niches for *P. gilberti* and *P. skiltonianus*. The first ratio *R*_rs_/*P*_rs_ is the range‐filling ratio (Svenning and Skov 2004) and is estimated from the correlative niche models. The remaining ratios are measured in multidimensional climate space and are based on the thermal niche measured from the mechanistic models (Monahan 2009)

	Range‐filling (*R*_rs_/*P*_rs_)	Monahan's *P*/*F*	Monahan's *R*/*P*
*P. gilberti*	0.69	0.35	0.41
*P. skiltonianus*	0.84	0.38	0.61

Classic multivariate statistical analyses revealed that *P. gilberti* and *P. lagunensis* were less diverged in niche space than either *P. lagunensis* and *P. skiltonianus* or *P. skiltonianus* and *P. gilberti*. DF1 and DF2 captured 68.3% and 31.7% of the variance in the dataset, respectively, and both functions had high canonical correlation values (DF1 = 0.77, DF2 = 0.63). DF1 is related to annual mean temperature and annual precipitation, while DF2 is related to precipitation variables (Table 5). Scatterplots of the two functions show that *P. gilberti* and *P*. *skiltonianus* separate along DF1, whereas *P. lagunensis* separated from the other two species along DF2. The discriminant analysis and jackknife test placed individuals in the correct species 87.4% and 87.0% of the time, respectively.

### Niche dynamics and distributions

The fundamental, potential, and realized thermal niches are all larger for *P. skiltonianus* than for *P. gilberti*. In both species, the fundamental thermal niche was larger than the potential niche and the potential niche was larger than the realized thermal niche, suggesting that factors other than critical physiological temperature shape the distributions of these species. Monahan's *P*/*F* ratio was similar for *P. skiltonianus* and *P. gilberti*, with low values indicating that both species are physiologically capable of colonizing a wider range of thermal conditions than is currently available near the ranges of either species (Table 7). In contrast, the *R*/*P* ratios were notably different; although the values for both species indicate that they are absent from regions with thermally suitable habitat, *P. gilberti* is absent from a greater proportion of its predicted range than *P. skiltonianus* (Table 7). In combination, these results suggest that abiotic and biotic factors other than critical thermal limits have shaped these species' ranges. These findings are in agreement with the range‐filling ratios (*R*_rs_/*P*_rs_) calculated from the climatic niche overlap analyses (Table 7). The range‐filling ratio indicates that neither species has filled the complete potential range predicted by climatic parameters; however, *P. skiltonianus* occupies a large portion of its potential niche, and apparently fills more of that niche than does *P. gilberti* (Table 7).

## Discussion

We quantify niche differences among morphospecies within the *P. skiltonianus* group and build on cumulative evidence in support of ecological speciation in these lizards. While these data alone do not identify the causes of speciation or provide a definitive test of adaptive evolution, they demonstrate that the morphospecies have significantly different ecologies.

They also represent a test of whether climate variables may have been involved in the divergence of a quantitative trait with clear implications on both thermal and aridity tolerance and reproductive isolation. This work also provides a foundation for clade‐specific, comparative analysis of niche evolution at smaller geographic scales, where heterogeneity in local environments may capture other key differences that allow ecological speciation to proceed to completion (Grant 1999; Soberón 2007).

### Are patterns of niche divergence consistent with thermally or climatically mediated ecological speciation?

Tests of niche equivalency and similarity based on Schoener's *D* statistic and Warren's *I* show that the climatic niches among members of the *P. skiltonianus* complex are more different than expected based on random predictions, and that the differences exceed those predicted by the background environments of the regions they inhabit (Tables 3, 4). Support for thermal adaptation as a contributor to speciation requires that temperature variables consistently explain most of the variation in these data. All of the niche differentiation tests show that temperature variables distinguish the niches of the different *P. skiltonianus* group members (Table 5). For example, annual mean temperature was an important variable underlying divergence in the McCormack's test, Broennimann's test, and the classical multivariate approaches. The combined temperature variables also explained over 70% of the variance in both of Broennimann's ordination methods. These findings are consistent with the outcome of thermal adaptation to different environments.

Some analyses further suggest that precipitation may be as or more important than temperature in determining distributional limits – correlative models identified precipitation as a distinguishing factor in the distributions of all three species (Table 1). The McCormack, Broennimann, and classic multivariate statistical approaches all showed evidence for niche divergence among precipitation variables (Table 5), and Maxent analyses indicated that four of the five parameters that best predict species' distributions involve precipitation. The two climate variables go hand in hand to some degree, especially in the more arid parts of the species' ranges in southwestern North America, and the importance of precipitation is not surprising given that these lizards are notoriously sensitive to desiccation (i.e., they associate with riparian areas near springs/creeks in the arid parts of the range, spend most of their time under surface litter, and tend to be active earlier in the year than most other lizards; Rodgers and Fitch 1947). Thus, in addition to critical temperatures, evaporative water loss and water retention may constitute an equally important, if not more important physiological constraint in these lizards, and suggests that both climatic and thermal niche differentiation may have contributed to ecological speciation in this system.

Evidence indicating significant affects of temperature and precipitation in distinguishing the niches of *P. skiltonianus* group members is consistent with an association between environmental differences and body size evolution, given that our comparisons were focused at the morphospecies level. Because size divergence has resulted in a mechanical reproductive barrier between morphospecies occurring in different environments (Richmond and Jockusch 2007; Richmond et al. 2011), it is possible that the barrier evolved as a by‐product of niche divergence. *Plestiodon gilberti* and *P. skiltonianus* are syntopic in parts of their range in California/Baja California, and niche overlap analyses predict greater co‐occurrence than the realized space. In some cases, areas of syntopy fall within regions of predicted overlap. Their ability to co‐occur may reflect microhabitat partitioning and possibly ecological character displacement (Costa et al. 2008). The two species are more differentiated in body size and juvenile color pattern where they are in closest contact in the southernmost parts of respective ranges, and overlap areas in southern California tend to be at the fringes of their respective local distributions (Stebbins 2003; Richmond 2005). The use of distribution models that can incorporate other sympatric lizard species that potentially compete with these skinks (alligator lizard *Elgaria multicarinata*, orange‐throated whiptail *Aspidoscelis hyperythra*) may also provide important insight into the distributional limits of *P. gilberti* and *P. skiltonianus* in these areas.

Definitive establishment of thermal and climate adaptation as a cause of speciation in the *P. skiltonianus* complex requires experimental testing on physiological tolerance and performance, and further comparative analysis of niche divergence in areas of syntopy. Nonetheless, the findings of this study provide ample evidence to justify further investigation of thermal adaptation as a by‐product mechanism leading to speciation in these lizards. Support for thermal adaptation as an underlying cause of ecological speciation is not well established, although a recent review identified 16 different empirical systems in which divergent selection across thermal gradients may have promoted speciation in a variety of taxa (Keller and Seehausen 2012). Thus, while limited evidence for this mechanism exists, the number of candidate cases and the diversity of taxa involved suggest that links between adaptive thermal divergence and the evolution of reproductive barriers may be more common than is currently understood.

### Niche divergence rather than niche conservatism promotes speciation of morphospecies

Climatic niche overlap scores generated for pairwise niche comparisons among species in the *P. skiltonianus* group were smaller than the null distribution in all comparisons, supporting the hypothesis of niche divergence over niche conservatism (Fig. 3). In general, critical thermal maxima are highly conserved across lizard lineages (with much of the variation accounted for by geography), whereas minima are less conserved (Grigg and Buckley 2013); however, we found that temperature maxima were more diverged than temperature minima in the *Plestiodon skiltonianus* complex (Table S2). This contrasts with the pattern found among *Liolaemus* lizards, where some species survive in the Andes Mountains at altitudes exceeding 4500 meters. *Liolaemus* are well known for having evolved adaptations for cold tolerance (Moreno Azócar et al. 2013), and perhaps the greater variation of temperature minima in this system is reflective of this history. By the same argument, the more divergent temperature maxima in the *P. skiltonianus* group may signal increased heat and aridity tolerance in *P. gilberti* lineages and is consistent with the predictions of the surface law. Furthermore, phylogenetic reconstruction of body morphology indicates that the larger *P. gilberti* morphology is derived (Richmond 2006), as expected if increased body size is adaptive.

It is important to note that the mechanistic model indicated that both the fundamental and potential niches were similar between the two morphospecies and that their primary difference reflected shifts in the realized niche (Fig. 2). It may be that the single parameter thermal niche captured by the models overlooks other important factors that would otherwise more precisely delimit their fundamental and potential niches. Specifically, precipitation is an equally or perhaps more important parameter not evaluated in our thermal models. Additional physiological data evaluating the effects of water loss and desiccation would be particularly informative.

### Factors aside from climate contribute to range dynamics

Although differences in niche space among members of the *P. skiltonianus* group are influenced by climatic conditions, the nonequivalency of the fundamental, potential, and realized niches indicates that factors aside from critical thermal tolerance also play a role in determining species' ranges. Furthermore, the degree of range restriction due to these factors is disproportionate with respect to each species (Table 7). For example, Svenning and Skov's *R*_rs_/*P*_rs_ ratio and Monahan's *R*/*P* ratio both indicate that the distribution of *P. gilberti* is more severely impacted by biotic interactions, dispersal limitations, or aspects of physiology than is *P. skiltonianus*.

Many of these impacts on *P. gilberti* relate to their low density on the Central Valley floor relative to surrounding areas, despite model predictions for widespread, suitable climate space in the Valley. Their lower density in this region is explained by the extensive replacement of natural lands by agriculture and urbanization, which restricts the species to small parcels of fragmented habitat (J. Q. Richmond, pers. obs.). In other parts of the *P. gilberti* range, physiogeographic barriers such as the Yuba River in the northern Sierra Nevada are clearly limiting the distributions of *P. gilberti* and *P. skiltonianus,* where the two species are restricted to opposite sides of the river despite consistency in the habitat on both sides (Shedd and Richmond 2013). Still in other parts of the range, older vicariant events are reflected in current phylogeographic patterns. For example, the geographic gap separating *P. lagunensis* in lower Baja California from a closely related *P. skiltonianus* lineage in northern Baja California/southern California may represent a historical vicariant event, as the same gap is observed in other vertebrate taxa (Riddle et al. 2000; Leaché et al. 2007).

Understanding the biological implications of these patterns and their relevance to speciation will require further study; however, they reiterate that factors other than climate are involved with interspecific divergence and that disentangling their relative contribution in causing versus maintaining the separation of lineages is not straightforward.

### Niche differentiation at the morphospecies vs. clade level: Are both informative?

Although the mechanism by which body size divergence causes reproductive isolation has already been identified for the *P. skiltonianus* group (Richmond and Jockusch 2007; Richmond et al. 2011), a key piece of missing information in confirming ecological speciation is the demonstration that body size increase represents an adaptive response to selection and that ecological divergence was the cause of lineage divergence. Ancestral reconstructions show that large body size is a derived state for this group, and at least some molecular evidence suggests that the large size has evolved repeatedly. The links between poikilothermy, the surface law, and possible phylogenetic signals of adaptation suggest that further studies on climatic or thermal adaptation as a cause of by‐product speciation are warranted.

A necessary part of testing for by‐product speciation includes statistical quantification of the niche space for members of the *P. skiltonianus* group. In this study, we used a top‐down approach to test for climate‐based niche differences by focusing at the morphospecies level and across the full distribution of each species. Measuring niche overlap and equivalency at this scale provides its own set of predictions for outcomes of ecological speciation, and consistent with these predictions, we found that the two predominant species' morphotypes have broadly differentiated niches. A logical extension of this work would test for similarities and differences at the clade level.

At the clade level, we would expect all *P. gilberti* clades to have largely overlapping niches (i.e., niche conservatism), assuming that similar environmental factors have driven the same evolutionary responses in separate clades. Similarly, we would expect the same pattern for clades of *P. skiltonianus*. The taxonomic (i.e., morphospecies vs. clade level) and geographic (rangewide vs. local) scale of this study may have also contributed to the detection of largely congruent fundamental and potential thermal niches for *P. skiltonianus* and *P. gilberti*, based on the mechanistic models involving critical thermal tolerance. The *P. skiltonianus* distribution spans an extremely broad range of latitude, longitude, and elevation, and nearly encompasses the entire *P. gilberti* range within it. Thus, while the scale of our approach here may have obfuscated some of the finer details of niche evolution in this group, we view it as a necessary first step and as a conservative test of the predicted outcomes of ecological speciation.

Depending on the analysis, signals of niche divergence between species were not always clear, nor were they always consistent with the expectations of the surface law (which predicts that the two small‐bodied species, *P. lagunensis* and *P. skiltonianus*, should show greater niche conservatism with respect to each other than either to *P. gilberti*). Niche dimensions unaccounted for likely explain some of these discrepancies; however, similar to the effects of scale, the resolution of the GIS data may have also introduced some bias in to our analyses, particularly in the characterization of the *P. lagunensis* niche. *Plestiodon lagunensis* occurs at southern latitudes in Baja California that far exceed the southern range limits of *P. skiltonianus* and *P. gilberti*, and populations are largely restricted to riparian areas near streams and springs in an otherwise desert landscape (Grismer 2002). If GIS data sources fail to capture important microclimate variation for this species at local scales, and instead describe more of the surrounding arid habitat, the *P. lagunensis* niche would appear more similar to *P*. *gilberti* than *P. skiltonianus* given the tendency for *P. gilberti* to occur in more xeric environments.

### Summary

While additional work is needed to definitively link body size evolution with divergent natural selection, this study has demonstrated that the niches of the two predominant, reproductively isolated morphs within the *P. skiltonianus* morph are significantly divergent and that the divergence cannot be explained by simple variability in the background environment. The overall pattern of niche divergence over niche conservatism, combined with clear evidence that both temperature and precipitation variables explain the major differences between the climate niches of the two morphospecies, lends support to the hypothesis that climate‐mediated adaptation is an important contributor to the speciation process in these lizards.

## Conflict of Interest

None declared.

## Supporting information


**Methods S1.** Niche Models and niche differentiation.
**Results S1.** Detailed results from paleo‐distributions and spatial overlap analyses.
**Results S2.** Detailed results from niche differentiation tests.
**Figure S1**. Niches and Niche Dynamics.
**Figure S2.** Correlative niche models current and paleo distributions.
**Figure S3**. Niche Overlap.
**Table S1.** Bioclim variables used in this study.
**Table S2.** Schoener's *D* and Niche Equivalency active months.

**Table S3.** Specimen data used in these analyses.
