# Supplementary material for: Niche divergence builds the case for ecological speciation in skinks of the Plestiodon skiltonianus species complex
Source: Ecol Evol. 2015 Oct 5;5(20):4683–95. doi: 10.1002/ece3.1610 (PMC4670058; doi:10.1002/ece3.1610)
Supplement: Supplementary file 1 — Methods S1. Niche Models and niche differentiation. Results S1. Detailed results from paleo‐distributions and spatial overlap analyses. Results S2. Detailed results from niche differentiation tests. Figure S1. Niches and Niche Dynamics. Figure S2. Correlative niche models current and paleo distributions. Figure S3. Niche Overlap. Table S1. Bioclim variables used in this study. Table S2. Schoener's D and Niche Equivalency active months. [file ECE3-5-4683-s001.docx]

Supplemental Material for:

Niche divergence builds the case for ecological speciation in skinks of the *Plestiodon skiltonianus* species complex

Guinevere O. U. Wogan and Jonathan Q. Richmond

# Contents page

Supplemental Methods S1 (Niche Models and niche differentiation) 2

Supplemental Results S1 (Detailed results from paleo-distributions and 3

spatial overlap analyses)

Supplemental Results S2 (Detailed results from niche differentiation tests) 4

Supplemental Table S1 (Bioclim variables used in this study) 6

Supplemental Table S2 (Schoener’s D and Niche Equivalency active months) 7

Supplemental Figure S1 (Niches and Niche Dynamics) 8

Supplemental Figure S2 (Correlative niche models current and paleo distributions) 9

Supplemental Figure S3 (Niche Overlap) 10

Supplemental References 11

**Supplemental Methods S1**

**Niches and niche models**

**Mechanistic Models.** The available measures in common for both species were CT_MAX_ (critical thermal maxima) and T_B_ (selected average temperature). Tests of critical thermal tolerance in *P. gilberti* ranged from a CT_MIN_ of 7.7°C to a CT_MAX_ of 42.3°C, with a T_B_ that ranged from 26.7 to 31.6°C (Brattstrom, 1965; Youssef *et al.*, 2008). For *P. skiltonianus* CT_MAX_ was 41.3°C (Brattstrom, 1965), with T_B_ values ranging from 25.2 − 30.0°C (Brattstrom, 1965; Cunningham, 1966). CT_MIN_ was available only for *P. gilberti (*7.7 °C), so we used the lowest recorded CT_MIN_ for *Plestiodon,* (measured in *P. chinensis*; Xu *et al.*, 1999) 6.2°C, as a lenient lower bound for *P. skiltonianus*. CT values in these studies were estimated using a standardized approach in which temperatures are changed incrementally and the lizard is flipped on its back. The temperature at which the lizard is no longer able to right iteslf is the critical thermal temperature.

Since these lizards are diurnal and T_MIN_ values generally reflect night-time temperatures, we used T_MEAN_ to set the lower climate boundary. Monthly T_MEAN_ and T_MAX_ data were obtained from WorldClim at 30 arc-second resolution (Hijmans *et al.*, 2005) − these are means averaged over a 50 year period, and should approximate long term environmental conditions. We ran these analyses for the lizard’s active period (March – June) and for the entire year. We present only those models from the lizard’s active period as the outcomes from these two analyses did not differ.

The suitable thermal habitat identified by the mechanistic models is the potential niche (the fundamental niche restricted by the realized climate). We generated 1000 random points within the potential niches of both species, and extracted the T_MEAN_ and T_MAX_ values. We then generated 5000 random points across the entire study region and extracted T_MEAN_ and T_MAX_ values to represent the realized climate of Western North America. We did the same (using 1000 points) within each of the IUCN distributions to represent the realized niche, and extracted T_MEAN_ and T_MAX_ values from museum specimen records. Records were obtained from the Museum of Vertebrate Zoology and the California Academy of Sciences, both of which have extensive collections for the *P. skiltonianus* complex (1467 unique localities; Figure 1) and most of which have been examined by JQR. Some bias may be introduced in theses measures due to incomplete sampling and the inclusion of data from juveniles. The IUCN maps capture the full range of each species, but also include local environments in which the lizard has not been documented.

**Correlative Niche Models.** The predicted distributions from contemporary and paleo-based models were thresholded using equal specificity and sensitivity (Figure S2), and stable areas were identified as those regions where species presence was predicted across 3 and 4 time periods. We use the predicted stable range to represent the core range in our estimates of spatial overlap.

**Niche Differentiation**

**Warren’s *I* and** ***D***. We tested for significant differences among species in *I* and *D* by generating probability distributions from 100 pseudoreplicate datasets of environmental data extracted from known species localities (Warren *et al.*, 2008). We rejected the hypothesis that niche overlap is a result of regional similarities or differences in available habitat if the inferred *I* and *D* values fell outside of the 95% confidence interval of these distributions (Warren *et al.*, 2008).

**McCormack’s autocorrelation method.** We developed the null model by performing principal components analysis (PCA) on the correlation matrix of climate variables from random points within the species’ distribution (i.e. the background environment - we used IUCN range polygons), as well as from the species occurrence points. The means of the extracted PC’s (eigenvalues greater than one) were treated as a composite ecological divergence score, and *T*-tests were used to compare species pairs. We calculated the mean of the background environment using a jackknife procedure of 750 samples with 1000 replicates, and then the absolute divergence between background areas was assessed by 1000 jackknife replicates of the means. We used the 95% density to delimit the null distribution of background environmental divergence, and compared this to the distribution of divergence in the realized niches.

**Broennimann’s method.** The first step of this test is to calculate the ‘smoothed’ densities of occurrence and of environmental factors along independent axes of a PCA, which ensures that any measurement of niche overlap is independent of the resolution of the grid data. Next, these smoothed data are pooled and randomly split into two data sets with the same number of occurrences as the original data set, and then niche overlap is measured using Schoener’s *D*. This process is repeated 100 times to produce a histogram of simulated values – if the observed *D* falls outside of the 95% density of the simulated values, the null hypothesis of niche equivalency is rejected. For the second test, random occurrence points from within one species range are used to generate the simulated data set, and overlap of the simulated niche with the observed niche in a second species’ range is again calculated using *D.* One hundred pseudoreplicates are used to produce a 95% density of simulated values – if the overlap with the observed data is less than 95% of the simulated values, the environments that the species occupies in the two ranges are significantly more different than expected by chance.

Because we were interested in testing whether certain climatic features can distinguish the three species, we used these same sets of analyses to evaluate the thermal minima, thermal maxima, and precipitation for each month during the active period (March – June). We used Worldclim and bioclim variables (Hijmans *et al.*, 2005) at 30 arc-second resolution for these analyses, reducing the number of re-sampled climate points to 0.05 degrees.

**Classic Multivariate Statistics.** We used multivariate analysis of variance (MANOVA) to quantify differences in the mean values of the 19 environmental variables among species. We calculated four *post hoc* MANOVA statistics: Wilks’lambda, Pillai’s trace, Lawley-Hotelling trace and Roy’s largest root. Significant results in MANOVA were followed by ANOVA tests for each of the individual variables. We used Discriminant Analysis to test whether the climate variables were able to predict species identities. Comparisons of Mahalonobis distances for each group were made using ANOVA, and we performed leave-one-out-cross-validation to test the classification strength of the model. We used Bonferroni correction for all multiple comparisons. **Supplemental Results S2**

**Paleo-Distributions**: Paleo-distribution models show considerable shifts in suitable climate space for members of the *P. skiltonianus* group over time. For *P.* *gilberti,* multiple expansions and contractions were revealed in association with Pleistocene climate change (Figure S1), the most dramatic being the range contraction away from the coast during the Last Glacial Maximum. However, during warmer periods such as the Last Interglacial, suitable climate space extended to the coast and substantially further north than it does at present. During the Last Glacial Maximum and the Last Interglacial period, the predicted range of *P. skiltonianus* was conspicuously reduced in the Sierra Nevada. The predicted range of *P. lagunensis* was restricted to extreme southern Baja Peninsula during the Last Interglacial Period, however during the Last Glacial Maximum, the predicted range expanded northward before once again contracting during the Holocene Hypsithermal.

**Overlap Analyses**: The correlative niche models predict overlapping ranges between *P. skiltonianus* and *P. lagunensis* along the coast of southern California and Baja California, and high overlap between *P. gilberti* and *P. lagunensis* in the Central Valley. *Plestiodon skiltonianus* and *P. gilberti* are predicted to overlap in portions of central California excluding the Central Valley, as well as Baja California. Predicted range overlap is lowest between *P. skiltonianus* and *P. lagunensis* and greatest between *P. gilberti* and *P. lagunensis* (Table 2). Overlap between the stable ranges of *P. gilberti* and *P. skiltonianus* is predicted in Northern California, the western edge of the Central Valley, throughout the Sierra Nevada and Transverse Ranges of southern California, and into northern Baja California (Figure S3).

The overlap among the distributions estimated from IUCN ranges, which we treat as a proxy for the real distribution, shows that the *P. skiltonianus* and *P. gilberti* overlap across 6.59% of their range as opposed to the 4.45% overlap predicted by the models. *Plestiodon lagunensis* does not overlap with either *P. gilberti* or *P. skiltonianus*, although the predictive models indicate 5.49% overlap and 0.04% overlap respectively (Table 2). The overlap estimated from the IUCN ranges for *P. gilberti* and *P. skiltonianus* extends along the western edge of the Central Valley and continues south into Baja California (Figure S2). The correlative niche models and the IUCN ranges for *P. gilberti* and *P. skiltonianus* both show high overlap along the western edge of the Central Valley and southward in to Baja California, however, the IUCN ranges have regions of overlap not identified in the correlative niche models and vice-versa. The observed ranges overlap extensively in California and over a small area in Nevada. The niche models predict extensive overlap in the Sierra Nevada as well as in Northern California (Figure S3).

Both *P. gilberti* and *P. skiltonianus* are observed within the areas of overlap predicted by the correlative niche models, particularly in southern California/Baja California and near the San Francisco Bay (Figure S3). Only *P. gilberti* is observed within the regions of predicted overlap in central California, whereas only *P. skiltonianus* occurs within the predicted overlap in northern California.

**McCormack’s Test:** Four of the 19 PCs had eigenvalues greater than one and were retained for use in McCormack’s Test. PC1 had high loadings for annual mean temperature (bio 1), mean temperature of the coldest month (bio 11), and minimum temperature of the coldest month (bio 6). PC2 had high loadings for precipitation of the wettest month (bio 13), precipitation of wettest quarter (bio 16), and temperature annual range (bio 7). PC3 had high loading for precipitation of the warmest quarter (bio18), mean temperature of driest quarter (bio 9), precipitation of coldest quarter (bio19), and PC4 for precipitation of warmest quarter (bio 18), precipitation of driest quarter (bio 17), and precipitation of driest month (bio14).

**Broennimann’s Test:** For PCA-env, PC1 related to annual mean temperature (Bio1) and mean temperature of the warmest quarter (Bio10). PC2 related to temperature annual range (Bio7) and temperature seasonality (Bio4). PCA-occ PC1 had high loadings for annual mean temperature (Bio1) and minimum temperature of the coldest month (Bio6), and PC2 for temperature seasonality (Bio4) and temperature annual range (Bio7). Analyses of thermal maxima and precipitation reveal that the niches of *P. gilberti* and *P. skiltonianus* are largely differentiated along these two climate parameters during the active months, while the niches are equivalent with respect to thermal minima (Table S2).

**Classic Multivariate Statistics:** All four of the *F*-statistic tests (Wilks’ Lambda, Pillai’ trace, Lawley-hotelling trace and Roy’s largest root) had significant P-values (*P* < 0.0001), indicating that at least one of the species group means differed in multivariate space, and ANOVA tests of the individual variables revealed that, the means were significantly different among species. We found that *P. gilberti* and *P. lagunensis* were less diverged in niche space than either *P. lagunensis* and *P. skiltonianus* or *P. skiltonianus* and *P. gilberti.* DF1 and DF2 captured 68.3% and 31.7% of the variance in the dataset, respectively and both functions had high canonical correlation values (DF1= 0.768, DF2= 0.633). DF1 had high loadings relating to annual mean temperature (bio 1) and annual mean precipitation (bio12). DF2 had the highest coefficients for precipitation of coldest quarter (bio19) and precipitation of wettest month (bio13). The comparison of Mahalanobis Distances of species means yielded significant *P*-values for all pairwise comparisons. Thus, the species’ means were significantly different in parameter space.

**Supplemental Table S1.** The 19 Bioclim variables used in this study (obtained from WorldClim at 30 arc second resolution).

| Bioclim 1 | Annual mean temperature |
| --- | --- |
| Bioclim 2 | Mean diurnal range |
| Bioclim 3 | Isothermality |
| Bioclim 4 | Temperature seasonality |
| Bioclim 5 | Max temperature of warmest month |
| Bioclim 6 | Min temperature of coldest month |
| Bioclim 7 | Temperature annual range |
| Bioclim 8 | Mean temperature of wettest quarter |
| Bioclim 9 | Mean temperature of driest quarter |
| Bioclim 10 | Mean temperature of warmest quarter |
| Bioclim 11 | Mean temperature of coldest quarter |
| Bioclim 12 | Annual precipitation |
| Bioclim 13 | Precipitation of wettest month |
| Bioclim 14 | Precipitation of driest month |
| Bioclim 15 | Precipitation seasonality |
| Bioclim 16 | Precipitation of wettest quarter |
| Bioclim 17 | Precipitation of driest quarter |
| Bioclim 18 | Precipitation of warmest quarter |
| Bioclim 19 | Precipitation of coldest quarter |

**Supplemental Table S2.** SDM-free niche quantitation using monthly thermal maxima, minima, and precipitation during the active period (March through June). Schoener’s D, and the P-value for niche equivalency are given. Statistically significant values at P<0.05 are in bold.

| WorldClim - monthly MaxTemp | Schoener’s D/  Niche Equivalency | WorldClim -  monthly MinTemp | Schoener’s D /  Niche Equivalency | World Clim-  monthly Precip | Schoener’s D /  Niche Equivalency |
| --- | --- | --- | --- | --- | --- |
| March | 0.84 / 0.93 | March | 0.82 / 0.52 | March | **0.64 / 0.02** |
| April | **0.73 / 0.02** | April | 0.92 / 0.16 | April | 0.76 / 0.24 |
| May | **0.69 / 0.02** | May | 0.91 / 0.28 | May | **0.64 / 0.02** |
| June | **0.69 / 0.04** | June | 0.76 / 0.18 | June | **0.62 / 0.04** |

**Supplemental Figure S1**

A. The interplay among realized, potential, and fundamental niche space (following Monahan 2009). The realized niche arises through limitations imposed by: (a) the potential niche (e.g. the organism has a broader tolerance of climate space than is currently available); (b) dispersal limitations (e.g. appropriate climate space exists, but the organism is unable to reach it due to physical barriers); and (c) biotic interactions (e.g. competitors prevent the focal taxon from occupying appropriate climate space, or other organisms upon which the focal taxon depends are not present). B. Four possibilities among niche models and actual ranges: (a) no predicted overlap of niches or taxa; (b) predicted overlap of niches but no overlap of taxa; (c) predicted overlap of niches and presence of one taxon; (d) predicted overlap of niches and presence of both taxa (Modified from Costa et al. 2008)


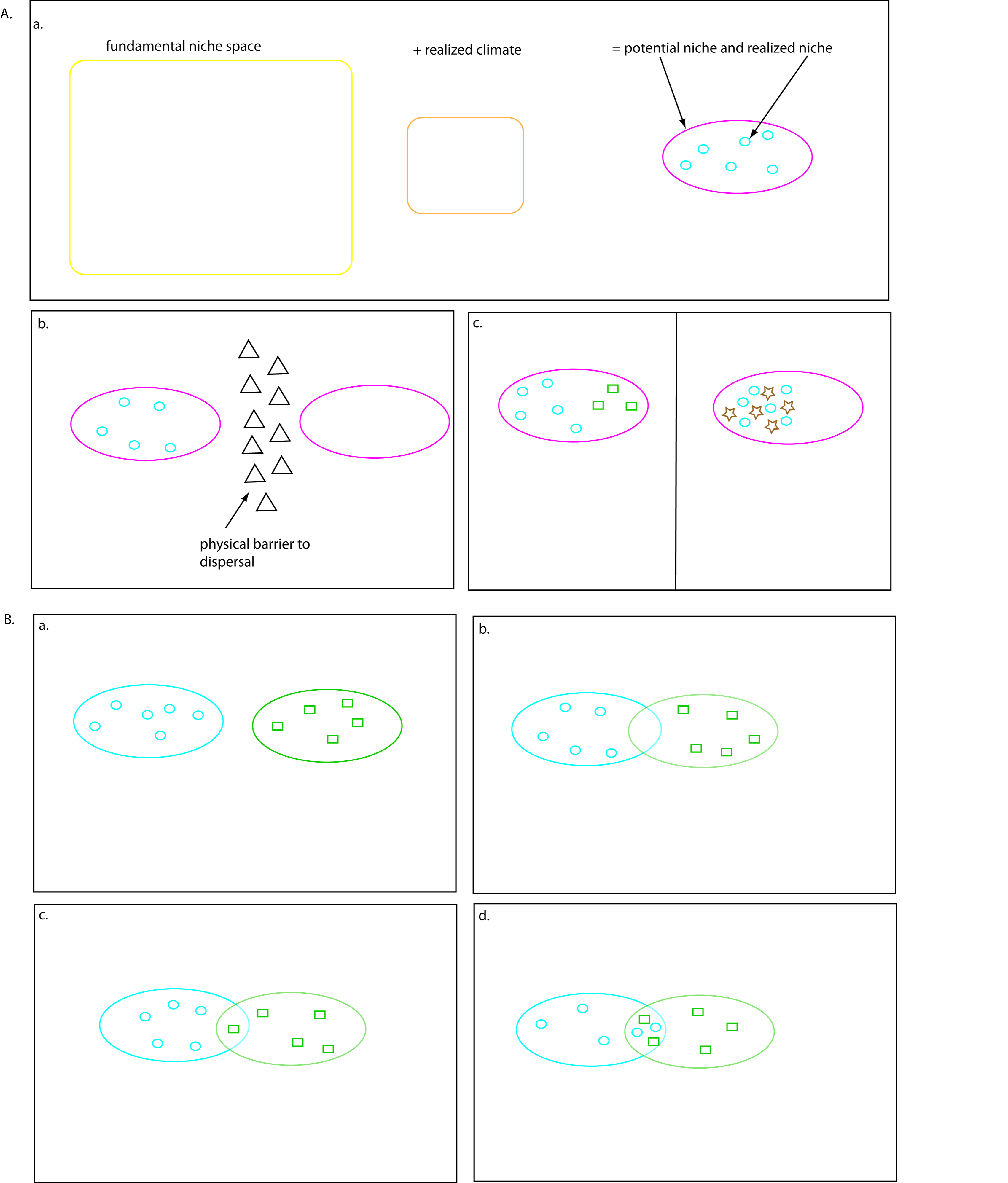


**Supplemental Figure S2**

Correlative models for each species under different climate regimes corresponding to the present, the Holocene hypsithermal (6 k.y.b.p.), the Last Glacial Maximum (21 k.y.b.p.), and the Last Interglacial Period (120 k.y.b.p.).


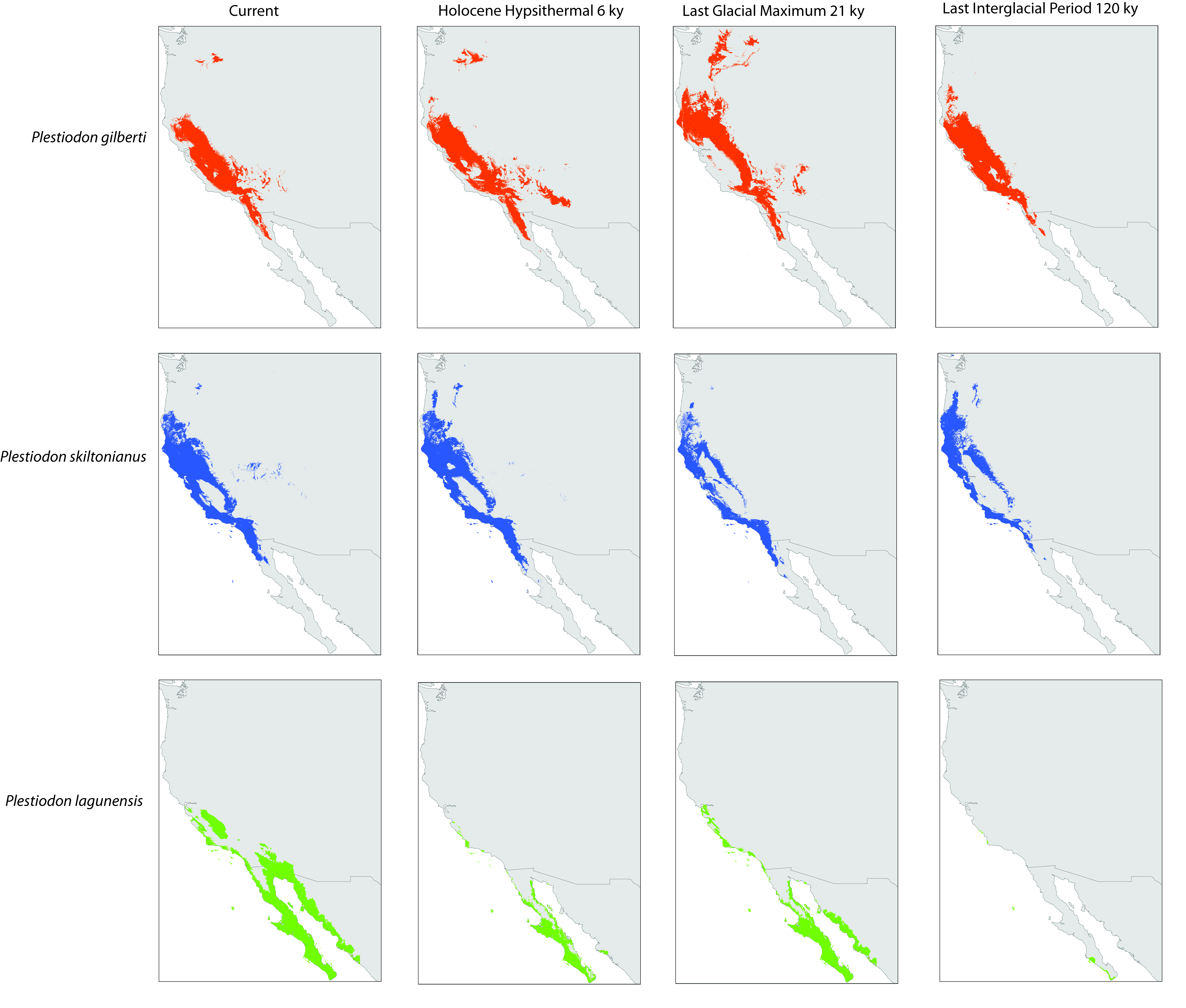


**Supplemental Figure S3**

Comparisons of range overlap for *P. gilberti* and *P. skiltonianus* using niche models (yellow) and IUCN distribution polygons (brown). Locations where niche models and IUCN polygons both predict range overlap are depicted in green.


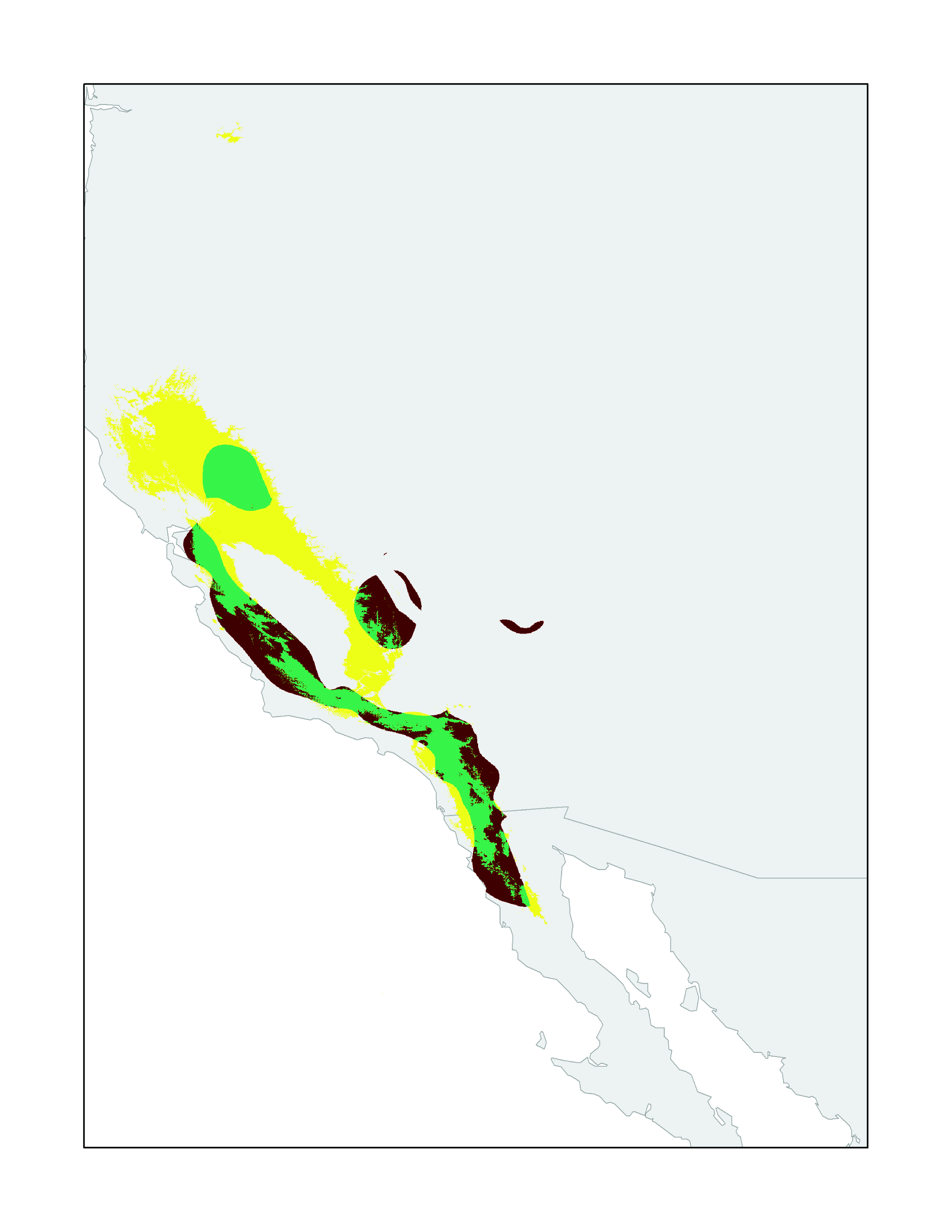


Supplemental References

Brattstrom, B. 1965. Body temperatures of reptiles. *American Midland Naturalist* **73:** 376-422.

Cunningham, J.D. 1966. Thermal relations of the alligator lizard *Gerrhonotus multicarinatus webbi*. *Herpetologica* **22:** 1-7.

Hijmans, R., Cameron, S.E., Parra, J.L., Jones, P.G. & Jarvis, A. 2005. Very high resolution interpolated climate surfaces for global land areas. *International Journal of Climatology* **25:** 1965-1978.

Warren, D.L., Glor, R.E. & Turelli, M. 2008. Environmental niche equivalency versus conservatism: quantitative approaches to niche evolution. *Evolution* **62:** 2868-2883.

Xu, X.F., Zhao, Q. & Ji, X. 1999. Selected body temperature, thermal tolerance and influence of temperature on food assimilation in juvenile Chinese skinks, *Eumeces chinensis* (Scincidae). *Raffles B Zool* **47:** 465-471.

Youssef, M.K., Adolph, S.C. & Richmond, J.Q. 2008. Evolutionarily conserved thermal biology across continents: The North American lizard *Plestiodon gilberti* (Scincidae) compared to Asian Plestiodon. *Journal of Thermal Biology* **33:** 308-312.
